# Impact of dose escalation on colostomy-free survival and treatment outcome in squamous cell anal carcinoma

**DOI:** 10.1007/s00066-023-02056-y

**Published:** 2023-03-02

**Authors:** Sebastian Untiedt, Daniel Rolf, Sergiu Scobioala, Heidi Wolters, Khaled Elsayad, Michael Oertel, Christopher Kittel, Andreas Pascher, Emile Rijcken, Hansjörg Ullerich, Bernhard Glasbrenner, Hans Theodor Eich

**Affiliations:** 1 Department of Radiation Oncology, University Hospital Muenster, 48149 Muenster, Germany; 2 Department for General, Visceral and Transplant Surgery, University Hospital Muenster, 48149 Muenster, Germany; 3 Department of Medicine B, Gastroenterology, University Hospital Muenster, 48149 Muenster, Germany; 4 Department of Medicine B, Gastroenterology, St. Franziskus-Hospital Muenster, 48145 Muenster, Germany

**Keywords:** Anal cancer, Radiotherapy, Radiation boost, Intensity-modulated radiotherapy, Toxicities

## Abstract

**Purpose:**

Primary radiochemotherapy (RCT) constitutes the standard of care for early- and advanced-stage anal carcinoma. This retrospective study investigates the impact of dose escalation on colostomy-free survival (CFS), overall survival (OS), locoregional control (LRC), progression-free survival (PFS), and acute and late toxicities in patients with squamous cell anal cancer.

**Methods:**

Considered were the outcomes of 87 patients with anal cancer treated with radiation/RCT between May 2004 and January 2020 at our institution. Toxicities were evaluated according to the Common Terminology Criteria for Adverse Events (CTCAE version 5.0).

**Results:**

The 87 patients received treatment with a median boost of 63 Gy to the primary tumor. With a median follow-up of 32 months, the 3‑year CFS, OS, LRC, and PFS were 79.5%, 71.4%, 83.9%, and 78.5%, respectively. Tumor relapse occurred in 13 patients (14.9%). Dose escalation to > 63 Gy (maximum 66.6 Gy) to the primary tumor in 38/87 patients revealed a nonsignificant trend for improved 3‑year CFS (82.4% vs. 97%, *P* = 0.092), a significantly improved CFS for T2/T3 tumors (72.6% vs. 100%, *P* = 0.008), and a significantly improved 3‑year PFS for T1/T2 tumors (76.7% vs. 100%, *P* = 0.035). While acute toxicities did not differ, dose escalation > 63 Gy led to a higher rate of chronic skin toxicities (43.8% vs. 69%, *P* = 0.042). Treatment with intensity-modulated radiotherapy (IMRT) showed a significant improvement in 3‑year OS (75.4% vs. 53.8%, *P* = 0.048). In multivariate analysis, significant improvements for T1/T2 tumors (CFS, OS, LRC, PFS), G1/2 tumors (PFS), and IMRT (OS) were shown. The nonsignificant trend for CFS improvement with dose escalation > 63 Gy was also apparent in multivariate analysis (*P* = 0.067).

**Conclusion:**

Dose escalation > 63 Gy (maximum 66.6 Gy) may improve CFS and PFS for certain subgroups, with a concomitant increase in chronic skin toxicities. Modern IMRT seems to be associated with an improvement in OS.

## Objective

Anal carcinoma is a rare malignant entity, the incidence and mortality of which have been increasing at both the national and international levels [1, 2]. Nigro et al. elaborated a neoadjuvant treatment regimen with combined radiochemotherapy (RCT) consisting of radiotherapy (RT) and simultaneous administration of 5‑fluorouracil (5-FU) and mitomycin C (MMC), which proved to be curative also in the definitive setting without surgery [3–5]. Consequently, concomitant RCT became the standard treatment for locally advanced anal canal carcinoma (LAACC) [6]. Further randomized trials underscored the superiority of 5‑FU and MMC as concomitant chemotherapy regarding reduction in local failure rate and increase in colostomy-free survival (CFS) in comparison to RT alone [7, 8] or in combination with single-agent 5‑FU [9]. However, the local recurrence and colostomy rates remained insufficient, with numbers reaching 36% [7], demanding of further improvements in treatment efficacy.

According to the German Cancer Consortium Radiation Oncology Group (DKTK-ROG), prognostic factors for disease-free survival (DFS) are classical clinicopathologic parameters like T category, N category, age, and Karnofsky performance score (KPS) [10].

One strategy to improve tumor control is RT dose escalation. Modern intensity-modulated radiotherapy (IMRT) has the potential to spare neighboring organs at risk while delivering increased doses to the target volume compared to 3D conformal radiotherapy (3D-CRT) [11–15]. IMRT leads to fewer toxicities than conventional RT techniques, not only in anal cancer but also in other tumor entities in the pelvic region [11, 12, 14, 16]. There are conflicting results on the optimal total dose, ranging between 45 and 70 Gy [7–9, 11, 17–22]. Retrospective analyses have reported improved local control (LC) with total doses ≥ 54 Gy [23–25]. Only one study found a trend toward a higher CFS rate (78% vs. 74%, *P* = 0.067) with an elevated tumor dose of 65–70 Gy vs. 60 Gy [19].

As a consequence, further data on this issue are needed to clarify the role of dose escalation. The present study investigates the influence of dose escalation in patients with anal cancer on colostomy-free survival (CFS), but also includes overall survival (OS), locoregional control (LRC), progression-free survival (PFS), and acute and late toxicities as secondary endpoints.

## Materials and methods

### Study design and data collection

The present analysis was designed as a single-institutional retrospective study including all patients with histologically proven anal squamous cell carcinoma treated with RT or RCT at our department between May 2004 and January 2020. Ethical approval was granted by our local institutional review board. Clinical data were collected via the electronic patient file as provided by our hospital information system (Orbis, Agfa Healthcare, Mortsel, Belgium), including medical reports, laboratory values, imaging, and follow-up notes. Additional data on RT details were provided by the information system of the department of radiation oncology (Aria, Varian Medical Systems, Pao Alto, CA, USA).

### Pretreatment assessment

All patients required a complete medical history, histologically confirmed diagnosis of anal cancer, physical examination, and laboratory evaluation. Systemic staging was performed according to the classification and staging system for anal cancer (AJCC 7th and AJCC 8th editions). Lymph node status was determined by computer tomography (CT) or sonography-based biopsy.

### Treatment techniques

All patients were discussed in the interdisciplinary tumor board to make a joint treatment decision. Furthermore, all patients underwent CT-based simulation, 44 patients had an additional planning magnetic resonance imaging (MRI), and 40 patients underwent additional positron-emission tomography-CT (PET-CT). The planning target volume (PTV) was delineated according to consensus contouring guidelines [26–28]. An IMRT treatment was received by 71 patients (81.6%), either linear accelerator based (38) or via tomotherapy (33), whereas 16 patients (18.4%) underwent 3D-CRT. All patients were treated with external beam RT. Sequential dose escalation (boost) to the primary tumor (PT) was applied in 78.2% (*n* = 68) of the patients, with a daily fraction of 1.8 Gy. An integrated boost technique was used in 11 patients (12.6%) and 8 patients (9.2%) received no boost at all. The RT boost was given immediately after completion of the larger pelvic fields, with no intended RT break unless an RT break was required due to toxicities. The median RT duration of this cohort was 52 days (range 21–85).

The concurrent chemotherapy regimen consisted of 5‑FU 1000 mg/m^2^ on days 1–4 and 29–32, and additional MMC 10 mg/m^2^ on days 1 and 29 according to the recommendation of the German S3 guideline for anal cancer [29]. A dose reduction of 5‑FU to 650 mg/m^2^ was possible for patients with decreased general and nutritional condition or previous heart disease.

### Follow-up

Follow-up visits were scheduled 2 months after RT and every 3–6 months thereafter. The oncologic surveillance included digital rectal examination with anoscopy and diagnostic imaging (pelvic CT/MRI) every 3–6 months. Abdominoperineal resection (APR) was recommended in patients with no change or disease progression at the primary location after pelvic RCT. Toxicities were assessed according to the Common Terminology Criteria for Adverse Events (CTCAE version 5.0) [30].

### Statistical analysis

All statistical analyses were conducted with IBM SPSS 27.0 software (IBM; Armonk, NY, USA). Colostomy-free survival (CFS) was calculated from the initiation of RT until colostomy for progression, relapse, or complication at the time of analysis. Overall survival (OS) was determined independently of the cause of death. Locoregional control (LRC) was calculated from initiation of RT until the time of relapse at the anal canal or margin, low rectum, vagina, pelvic or inguinal area. Progression-free survival (PFS) was calculated from the initiation of RT until documented locoregional or extrapelvic relapse or death. Survival data were analyzed using the Kaplan–Meier event curves and compared with a log-rank test. Variables associated with CFS, OS, LRC, or PFS in univariate analysis (*p* < 0.1) were entered into a Cox proportional hazard regression model for multivariate analysis. Chi-squared or Fisher exact tests were used to analyze the relationship between two categorial variables. Differences were considered statistically significant at a *P*-value < 0.05.

## Results

In total, 87 patients (57 female, 30 male) with anal cancer were treated at our institution. Baseline patient, tumor, and treatment characteristics are reported in Table 1. With a median follow-up period of 32 months (range 0–179 months), the 3‑year CFS, OS, LRC, and PFS were 79.5%, 71.4%, 83.9%, and 78.5%, respectively. Tumor relapse occurred in 13 patients (14.9%), including 5 locoregional relapses (5.7%; local relapse and/or regional lymph node metastases), 4 distant recurrences (4.6%), and 4 patients who had both.

**Table 1:** Patient, tumor, and treatment characteristics

	*N*	Percentage/range
**Patient characteristics**		
*No. patients*	87	–
*Median age at diagnosis, years*	59	34–88
*Gender*		
Male	30	34.5%
Female	57	65.5%
*Previous malignancy*		
Yes	14	16.1%
No	73	83.9%
**Tumor characteristics**		
*Histology*		
Squamous cell carcinoma	87	100%
*Localization*		
Anal canal	71	81.6%
Anal margin	16	18.4%
*Median tumor maximum diameter, cm*	4.0	0.4–10.0
*T classification*		
T1	21	24.1%
T2	31	35.6%
T3	22	25.3%
T4	10	11.5%
Unknown	3	3.5%
*N classification*		
N0	50	57.5%
N +	36	41.4%
Unknown	1	1.1%
*M classification*		
M0	84	96.6%
M1	2	2.3%
Unknown	1	1.1%
*Grading*		
G1/G2	55	63.2%
G3	29	33.3%
Unknown	3	3.5%
**Treatment characteristics**		
*Pre-RT-MRI*	44/87	50.6%
*Pre-RT-PET*	40/87	46%
*RT technique*		
IMRT	71	81.6%
Conventional (3D-CRT)	16	18.4%
*RT lymphatic drainage pathway*		
Median boost dose, Gy	59.1	50.4–66.6
*RT primary tumor*		
Median boost dose, Gy	63.0	35.2–66.6
*Boost technique*		
Sequential boost	68	78.2%
Integrated boost	11	12.6%
No boost	8	9.2%
*Median boost volume, cm*^*3*^	241.1	42.3–1444.9
*Median RT duration, days*	52	21–85
*Chemotherapy*		
MMC +5-FU	70	80.5%
MMC +5-FU reduced dose	8	9.2%
None	9	10.3%
*Median follow-up, months*	32	0–179
*Colostomy*	16/87	18.4%
***Relapse pattern***		
*Yes*	13	14.9%
Locoregional only	5	5.7%
Distant only	4	4.6%
Both	4	4.6%
*No*	55	63.2%
*Unknown*	19	21.9%

*RT* radiotherapy, *MRI* magnetic resonance imaging, *PET* positron-emission tomography, *IMRT* intensity-modulated radiotherapy, *MMC* mitomycin C, *5‑FU* 5-fluorouracil

Median boost treatment volume was 241.1 cm^3^. We stratified the boost target volumes according to this value and found a nonsignificant trend towards a lower RT boost volume with the use of PET-CT planning (≤ 241.1 cm^3^ vs. > 241.1 cm^3^; *P* = 0.197). Treatment with intensity-modulated radiotherapy showed a significant improvement in 3‑year OS (IMRT vs. 3D-CRT: OS 75.4% vs. 53.8%, *P* = 0.048), but there were no significant differences between IMRT and 3D-CRT for CFS (*P* = 0.235), LRC (*P* = 0.209), and PFS (*P* = 0.139). There was an improved outcome regarding 3‑year CFS, OS, LRC, and PFS for early-stage (T1/T2) tumors in comparison to more advanced stage tumors (T3/T4; T1/T2 vs. T3/T4: CFS 91.1% vs. 63.6%, *P* < 0.001; OS 88.8% vs. 43.5%, *P* < 0.001; LRC 94.6% vs. 63.9%, *P* = 0.003; PFS 88% vs. 60.5%, *P* = 0.028). Regarding the lymph node status, there was an improved 3‑year OS for patients with N0 compared to *N* + (84.6% vs. 52%, *P* = 0.027). Furthermore, an improvement in 3‑year PFS was shown for G1/2 tumors (G1/2 vs. G3: PFS 86.2% vs. 62.7%, *P* = 0.021).

### Dose escalation

**Fig. 1:**
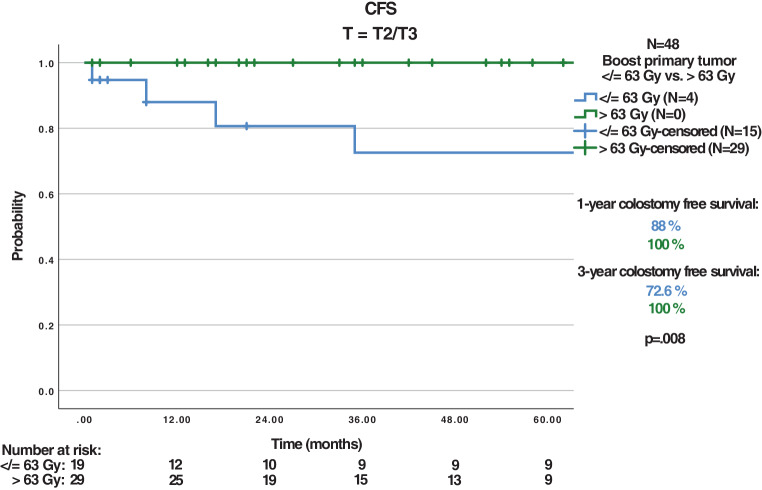
Colostomy-free survival (*CFS*) T2/T3 with dose escalation ≤ 63 Gy vs. > 63 Gy

**Fig. 2:**
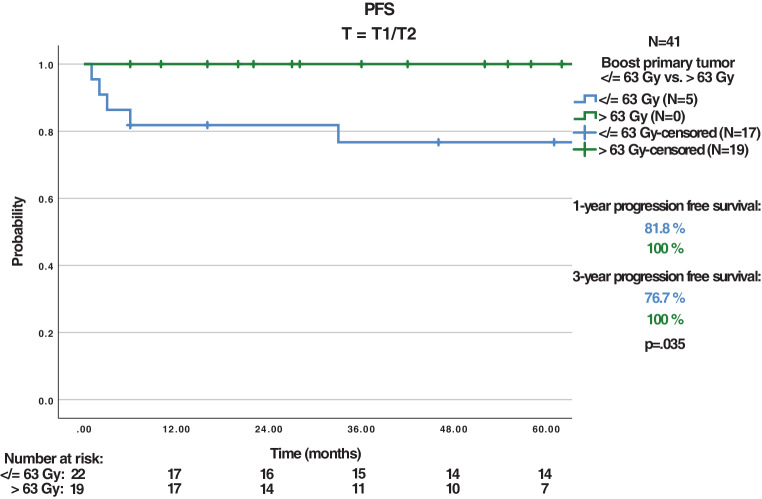
Progression-free survival (*PSF*) T1/T2 with dose escalation ≤ 63 Gy vs. > 63 Gy

The median RT boost dose regarding the primary tumor (PT) was 63 Gy (range 35.2–66.6) and 59.1 Gy (range 50.4–66.6) for the lymphatic drainage pathways (LDP). Some patients received a lower total radiation dose: one patient discontinued the radiation against medical advice at a total dose of 35.2 Gy due to mental illness. Because of previous infield irradiation, one patient was irradiated with a dose of 36 Gy. Due to old age and multiple secondary diseases, two patients were irradiated with a total dose of 39.8 Gy and 45 Gy. These patients were excluded from the analysis of dose escalation. Median RT boost volume was 241.1 cm^3^ (range 42.3–1444.9). A dose escalation to the PT > 63 Gy (≤ 63 Gy vs. > 63 Gy; maximum 66.6 Gy) did not influence 3‑year OS (*P* = 0.812), LRC (*P* = 0.587), or PFS (*P* = 0.305); however, a nonsignificant trend towards improved 3‑year colostomy-free survival (82.4% vs. 97%, *P* = 0.092) could be observed. A subgroup analysis of the patients with a dose escalation > 63 Gy revealed a significantly improved 3‑year CFS in patients with T2/T3 tumors (72.6% vs. 100%, *P* = 0.008) and a significantly improved 3‑year PFS in patients with T1/T2 (76.7% vs. 100%, *P* = 0.035) tumors (Fig. 1&2).

Using the median RT boost dose to the LDP (≤ 59.1 Gy vs. > 59.1 Gy) as a cut-off, no prognostic advantages resulted from high-dose RT (CFS *P* = 0.578; OS *P* = 0.483; LRC *P* = 0.503; PFS *P* = 0.594).

### Cox proportional hazard model

Univariate analysis (Table 2) suggests that the T status and RT boost dose to the primary tumor may impact CFS. Furthermore, age, T and N status, radiation technique (IMRT vs. 3D-CRT), and boost volume may influence OS. The T status seems to have an effect on LRC and PFS, whereas grading may be a predictor of PFS. The multivariate analysis (Table 2) for CFS revealed a significant improvement for T1/T2 tumors (HR 5.23, *P* = 0.005) and a nonsignificant trend for an improvement with dose escalation > 63 Gy (HR 0.342, *P* = 0.067). Furthermore, the multivariate analysis showed a significantly improved OS for T1/T2 tumors (HR 6.37, *P* = 0.008) and IMRT technique (HR 3.83, *P* = 0.024), while T1/T2 tumors (HR 4.07, *P* = 0.021) and G1/2 tumors (HR 4.49, *P* = 0.015) showed significant advantages for PFS.

**Table 2:** Univariate and multivariate analyses for colostomy-free survival (CFS), overall survival (OS), locoregional control (LRC), and progression-free survival (PFS)

Variable	CFS		OS		LRC		PFS	
	HR	*p*-value	HR	*p*-value	HR	*p*-value	HR	*p*-value
*Univariate model*								
Age (≤ 59 years vs. > 59 years)	0.711	0.509	1.89	0.089	1.0	> 0.999	0.767	0.642
Gender (female vs. male)	1.99	0.170	1.77	0.124	2.21	0.240	2.12	0.178
T status (T1/T2 vs. T3/T4)	5.53	0.004	5.10	< 0.001	8.10	0.011	3.40	0.038
N status (N0 vs. *N* +)	1.98	0.176	2.22	0.032	2.28	0.221	2.03	0.205
Grading (G1/2 vs. G3)	1.20	0.731	1.72	0.147	1.59	0.490	3.44	0.030
Pre-RT-MRI	1.65	0.333	1.10	0.806	1.75	0.429	1.44	0.523
Pre-RT-PET	1.09	0.867	1.57	0.239	3.28	0.138	1.97	0.258
IMRT vs. conventional	1.08	0.910	2.13	0.048	0.039	0.425	0.039	0.346
Boost LDP (≤ 59.1 Gy vs. > 59.1 Gy)	1.02	0.985	79.58	0.161	2.22	0.514	1.62	0.597
Boost primary tumor (≤ 63 Gy vs. > 63 Gy)	0.374	0.092	1.10	0.813	0.674	0.590	0.539	0.313
Boost volume (≤ 241.1 cm^3^ vs. > 241.1 cm^3^)	2.82	0.110	2.50	0.059	2.35	0.244	1.80	0.316
*Multivariate model*								
Age (≤ 59 years vs. > 59 years)	–	–	1.57	0.326	–	–	–	–
T status (T1/T2 vs. T3/T4)	5.23	0.005	6.37	0.008	8.10	0.011	4.07	0.021
N status (N0 vs. N +)	–	–	1.16	0.804	–	–	–	–
Grading (G1/2 vs. G3)	–	–	–	–	–	–	4.49	0.015
IMRT vs. conventional	–	–	3.83	0.024	–	–	–	–
Boost primary tumor (≤ 63 Gy vs. > 63 Gy)	0.342	0.067	–	–	–	–	–	–
Boost volume (≤ 241.1 cm^3^ vs. > 241.1 cm^3^)	–	–	0.963	0.951	–	–	–	–

*RT* radiotherapy, *MRI* magnetic resonance imaging, *PET* positron emission tomography, *IMRT* intensity-modulated radiotherapy, *LDP* lymphatic drainage pathway

### Toxicities

The acute adverse events with their maximum grading are summarized in Table 3. During RT, almost all patients developed acute grade 1 (97.7%) and grade 2 (93.1%) adverse events, while grade 3 and grade 4 toxicities were observed in 42.5% and 6.9% of the patients, respectively. The most common acute toxicities affected the skin (93.1%), were hematologic disorders (91.9%), and affected the gastrointestinal tract (90.8%). Comparing the RT boost dose to the PT (≤ 63 Gy vs. > 63 Gy), no significant differences could be determined. No patient required APR for reasons of acute toxicity. Chronic adverse events are summarized in Table 3. The most common chronic toxicities concerned the gastrointestinal tract (46%) and the skin (39.1%). Dose escalation higher than 63 Gy led to a higher rate of chronic skin toxicities (≤ 63 Gy vs. > 63 Gy; 43.8% vs. 69%, *P* = 0.042). No significant differences were observed comparing the RT boost dose to the LDP (≤ 59.1 Gy vs. > 59.1 Gy) in terms of acute or chronic adverse events.

**Table 3:** Acute and chronic toxicities

*Acute toxicity (max)*	*Grade 1*	*Grade 2*	*Grade 3*	*Grade 4*
Skin	26 (29.9%)	52 (59.8%)	3 (3.4%)	0 (0%)
Gastrointestinal	21 (24.1%)	44 (50.6%)	14 (16.1%)	0 (0%)
Hematologic	27 (31%)	26 (29.9%)	21 (24.1%)	6 (6.9%)
Cardiac	0 (0%)	2 (2.3%)	3 (3.4%)	0 (0%)
General	17 (19.5%)	22 (25.3%)	2 (2.3%)	0 (0%)
Urinary	31 (35.6%)	0 (0%)	0 (0%)	0 (0%)
Vascular	1 (1.1%)	0 (0%)	0 (0%)	0 (0%)
Reproductive system	1 (1.1%)	1 (1.1%)	0 (0%)	0 (0%)
*Chronic toxicity*	*N*	*Percentage*		
Skin	34	39.1%		
Gastrointestinal	40	46%		
Hematologic	0	0%		
Cardiac	0	0%		
General	10	11.5%		
Urinary	2	2.3%		
Vascular	4	4.6%		
Reproductive system	12	13.8%		

## Discussion

This study presents the survival outcomes and toxicities of 87 patients with anal cancer, treated with definitive radiation/chemoradiation at our institution. The following key findings emerged from this study:

1. Trend for improved CFS and significant improvement for T2/T3 tumors with dose escalation > 63 Gy.
2. Improved PFS with dose escalation > 63 Gy for T1/T2 tumors.
3. Trend towards smaller boost volume with PET-CT planning.
4. Improved OS with IMRT treatment compared to conventional techniques (3D-CRT).

The dose of the radiation boost is controversial and there is a lack of recommendations concerning the optimal tumor dose, resulting in a variety of concepts with RT doses between 45 and 70 Gy [7–9, 11, 17–22]. There is only retrospective evidence for the comparison of RCT with brachytherapy boost and a boost using external beam radiation [31–34].

Surrogate parameters like LC and PFS have been used to assess the efficacy of dose escalation and treatment intensification in previous trials. CFS was also the primary endpoint in the LAACC study of the European Organisation for Research and Treatment of Cancer (EORTC) [8] and ACCORD 03 [19], and is influenced by LC as well as by the absence of high-grade toxicities. The cumulative total doses used in the latter studies were quite similar to those used in the current study, with a median boost dose to the PT of 63 Gy. Bartelink et al. [8] showed a significant improvement of CFS in patients with combined radiochemotherapy vs. radiotherapy alone. The prospective randomized ACCORD 03 [19] trial revealed a nonsignificant trend for improved 3‑ and 5‑year CFS with a high-dose (HD) boost (HD 79% and 77.8% vs. standard dose [SD] 76% and 73.7%, respectively; *P* = 0.067). Correspondingly, the present analysis displays an, albeit not significant, improvement in CFS and a significant amelioration for T2/T3 tumors with dose escalation > 63–66.6 Gy. However, the number of patients (*n* = 48) in this subgroup analysis is relatively small and other retrospective analyses deny this association [35, 36]. Overall, the reported CFS ranges between 68 and 84.3% at 3 years, so that the CFS of the entire cohort in this current study was in the upper range with a 3-year CFS of 79.5% [19, 35–39]. Considering only the patients who received a high-dose boost > 63 Gy, this study showed a superior 3‑year CFS of 97%. The aforementioned ACCORD 03 [19] trial also investigated LC and showed a small nonsignificant improvement for LC at 3 and 5 years with a high-dose boost (HD 84%, 83.1% vs. SD 79%, 78.2%; *P* = 0.28). In accordance with other studies, the locoregional control of the entire cohort was high, with 93.6% and 83.9% at 1 and 3 years [36, 40, 41]. As described above, CFS reflects the combination of LC and the absence of severe toxicities [19]. Overall, the number of complete remissions could be higher in dose-escalated patients, leading to a lower locoregional recurrence rate and reducing the necessity for colostomies. However, we could not detect any significant difference between higher or lower radiation doses regarding LRC (82.5% vs. 86.7%, *P* = 0.587), and the therapeutic benefit must be weighed up against an increased rate of chronic skin side effects. While the ACCORD 03 [19] trial showed a nonsignificant trend towards improved CFS, this study showed significant improved CFS at 3 years for T2/T3 tumors with dose escalation > 63 Gy. This merits further investigations.

Dose-escalated RT may also improve the prognosis of small (T1/T2) tumors, with a dose escalation > 63 Gy resulting in longer PFS. The herein reported PFS rate of 78.5% at 3 years is high in comparison to the literature, which reports 3‑year PFS values of 67–80.2% [18, 19, 35–39, 42, 43]. In this study, patients with dose escalation > 63 Gy and T1/T2 tumors showed significantly improved 3‑year PFS (76.7% vs. 100%, *P* = 0.035), and by limiting the analysis to patients who received a high-dose boost > 63 Gy, 3‑year PFS even reached 83.7%. However, a similar attempt in another retrospective analysis which employed RT doses beyond 54 Gy to improve PFS was unsuccessful [35]. Furthermore, it should be considered that the number of patients (*n* = 41) in this subgroup analysis of patients with T1/T2 tumors and dose escalation > 63 Gy is relatively small. In terms of acute toxicities, this study revealed no significant differences between different RT boost doses (≤ 63 Gy vs. > 63 Gy): 21 patients with a cumulative total dose ≤ 63 Gy showed ≥ grade 3 toxicities, whereas 20 patients with a high dose boost > 63 Gy had ≥ grade 3 toxicities. However, a significant higher rate of chronic skin toxicities with a dose escalation > 63 Gy could be observed (43.8% vs. 69%, *P* = 0.042). The spectrum and frequency of acute and chronic toxicities is inhomogeneous in the literature, hampering direct comparisons [11, 18, 24, 36, 37, 39, 40, 44, 45]. Considering the relatively high RT doses used in this analysis, the toxicities were mostly tolerable, despite the higher rate of chronic skin toxicities.

A potential approach to improving the outcome of patients with anal cancer is RCT combined with deep regional hyperthermia as a radiosensitizer. Kouloulias et al. [46] randomized 49 patients with T2-3N0M0 anal cancer into two study arms. In both arms, patients received RT with 41.4 Gy (1.8 Gy per fraction) and a dose escalation of 14 Gy (2 Gy per fraction) to a total dose of 55.4 Gy, and concomitant chemotherapy consisting of 5‑FU and MMC. Arm A (*n* = 24) also received an intracavitary hyperthermia treatment once a week in six sessions. Kouloulias et al. [46] showed in their study that 23/24 (95.8%) patients with concomitant hyperthermia treatment retained their anorectal function and avoided permanent colostomy, whereas only 17/25 (68%) patients without hyperthermia treatment showed sphincter preservation. Furthermore, the 5‑year local recurrence-free survival was significantly higher with hyperthermia (59.7% vs. 50.4%, *P* = 0.0107); however, there were no significant advantages in terms of OS. In another study by Ott et al. [47] with 112 patients, all patients received RT (55.8–59.4 Gy) with concomitant chemotherapy consisting of 5‑FU and MMC. Fifty of the patients also received an additional deep regional hyperthermia treatment. After a follow-up of 5 years, the group that additionally received hyperthermia showed improvements in OS (95.8% vs. 74.5%, *P* = 0.045), disease-free survival (89.1% vs. 70.4%, *P* = 0.027), local recurrence-free survival (97.7% vs. 78.7%, *P* = 0.006), and CFS (87.7% vs. 69%, *P* = 0.016). They also reported that the additional hyperthermia did not increase acute and late toxicities except for hematotoxicity (66% vs. 43%, *P* = 0.032) and the rate of telangiectasia (38% vs. 16.1%, *P* = 0.009), which were higher in the group with hyperthermia treatment. Therefore, the prospective HyCAN trial currently running in Germany (ClinicalTrials.gov Identifier: NCT02369939) investigates the impact of deep regional hyperthermia in addition to RT (T2N0: 55.8 Gy, T3-4N0: 59.4 Gy) and concomitant chemotherapy with 5‑FU and MMC.

Another finding was a nonsignificant trend towards a lower RT boost volume (≤ 241.1 cm^3^ vs. > 241.1 cm^3^; *P* = 0.197) in patients with PET-CT planning. PET-CT may serve as a further diagnostic tool to precisely confirm the location of the PT and detect affected/unaffected lymph nodes, thus influencing staging and target volume delineation [48–54]. In a study by Zimmermann et al. [51], upstaging and downstaging by PET-CT were reported in 13% of cases each for nodal disease, with a consequent change in treatment planning for 17% of the patients. A systematic literature review by Mahmud [52] showed that PET-CT upstaged 5.1–37.5% and downstaged 8.2–26.7% of patients with anal cancer and the treatment plans were revised in 12.5–59.3%. In addition, a sensitivity of 99% for PET-CT and 67% for CT in the detection of PT was reported, as well as an overall sensitivity of 93% and specificity of 76% for PET-CT in the detection of inguinal lymph nodes. Current studies on motion-compensated PET-CT are available for other tumor localizations such as esophageal cancer, but this is not of great relevance for anal cancer due to the low mobility in the anal area [55]. As a limitation of the current study, more patients in an early stage of disease than in an advanced stage received a PET-CT for RT planning. Patients of this cohort in early stages also had a boost volume below the median more often than patients in advanced stages.

The apparent survival improvement with IMRT treatment (3-year OS IMRT vs. 3D-CRT; 75.4% vs. 53.8%; *P* = 0.048) is in accordance with other studies [12, 56, 57]. The distributions of early and advanced stages of disease were homogeneous in the IMRT group as well as in the 3D-CRT group. Nevertheless, only 18.4% of the study cohort were treated with 3D-CRT and may have been subject to bias due to the low number of patients. We hypothesize that IMRT creates a more homogeneous distribution of the radiation doses, and higher applied RT doses, as in our cohort, combined with reduced nonhematologic toxicities [11, 12, 14], may possibly lead to improved OS.

It remains unclear which RT dose is optimal at which stage of the disease and results from ongoing studies are awaited. The ongoing PLATO trials (ACT 3, ACT 4, and ACT 5) investigate the optimal RT dose in terms of dose de-escalation in early stages of disease and dose escalation in locally advanced disease (ISRCTN88455282).

## Limitations

This study has several limitations intrinsic to its retrospective and monoinstitutional character. Confounding cannot be excluded and is a possible disruptive factor. This study is based on only 87 patients and the distribution within the individual groups was not always equal. Furthermore, another limitation is the lack of some data from various variables.

## Conclusion

The results suggest dose escalation > 63 Gy (maximum 66.6 Gy) as a potential suitable instrument to improve CFS and PFS for certain subgroups. The higher efficacy has to be balanced carefully against an increased rate of chronic skin toxicities. PET-CT, as an additional imaging modality, may enable more precise delineation of the target volume and allow the contouring of smaller boost volumes. Modern IMRT seems to be associated with an improvement in OS.
